# Immune-Based Approaches for the Treatment of Pediatric Malignancies

**DOI:** 10.1146/annurev-cancerbio-030419-033436

**Published:** 2020-03

**Authors:** Kristopher R. Bosse, Robbie G. Majzner, Crystal L. Mackall, John M. Maris

**Affiliations:** 1 Division of Oncology and Center for Childhood Cancer Research, Children’s Hospital of Philadelphia, Philadelphia, Pennsylvania 19104, USA; 2 Department of Pediatrics, Perelman School of Medicine, University of Pennsylvania, Philadelphia, Pennsylvania 19104, USA; 3 Department of Pediatrics and Stanford Cancer Institute, Stanford University School of Medicine, Stanford, California 94305, USA

**Keywords:** pediatric cancers, immunotherapy, antibodies, CAR T cells

## Abstract

Immune-based therapies have now been credentialed for pediatric cancers with the robust efficacy of chimeric antigen receptor (CAR) T cells for pediatric B cell acute lymphocytic leukemia (ALL), offering a chance of a cure for children with previously lethal disease and a potentially more targeted therapy to limit treatment-related morbidities. The developmental origins of most pediatric cancers make them ideal targets for immune-based therapies that capitalize on the differential expression of lineage-specific cell surface molecules such as antibodies, antibody-drug conjugates, or CAR T cells, while the efficacy of other therapies that depend on tumor immunogenicity such as immune checkpoint inhibitors has been limited to date. Here we review the current status of immune-based therapies for childhood cancers, discuss challenges to developing immunotherapeutics for these diseases, and outline future directions of pediatric immunotherapy discovery and development.

## INTRODUCTION

Pediatric cancers are fundamentally different than adult tumors in that they arise from a misappropriation of normal developmental processes rather than developing in response to decades of environmentally mediated DNA damage, as is typical in adult malignancies (Chun et al. 2016, Mosse et al. 2008). As a result of these developmental origins, tumors in children have a significantly lower mutation burden than in adults (Chalmers et al. 2017), typically containing only a limited number of driver genetic alterations such as biallelic *INI1* loss in malignant rhabdoid tumors (Chun et al. 2016), *RB1* mutations in retinoblastoma (Friend et al. 1986), *MYCN* amplification and *ALK* (anaplastic lymphoma kinase) mutations in neuroblastoma (Mosse et al. 2008, Schwab et al. 1983), *H3K27M* mutations in diffuse intrinsic pontine gliomas (Wu et al. 2012), or *EWSR1-FLI1* or *PAX3-FOXO1* translocations in Ewing sarcoma (Ohno et al. 1994) and rhabdomyosarcoma (Galili et al. 1993), respectively. Consequently, childhood tumors do not contain an abundance of recurrent and clinically targetable mutated oncogenes, despite sharing many of the same driver mutations, albeit at much lower frequencies (Chalmers et al. 2017, Grobner et al. 2018, Ma et al. 2018). Thus, to date pediatric cancer treatment approaches have been limited to empiric cytotoxic chemoradiotherapies that come with a myriad of immediate and late life-threatening treatment-related comorbidities. While decades of clinical research focused on safely and effectively combining these therapies have resulted in improved clinical outcomes, still today many children ultimately have refractory disease or are afflicted with lifelong morbidities from their treatments (Oeffinger et al. 2006). Furthermore, most children with relapsed cancers remain incurable with our current treatment regimens.

Over the last decade, the ability to capitalize on the antitumor capabilities of the host immune system has revolutionized cancer treatment approaches, with major breakthroughs in childhood leukemias (Majzner et al. 2017). Furthermore, because resistance to standard cytotoxic agents does not imply resistance to immunotherapeutics and since toxicities of immunotherapy are noncumulative and nonoverlapping with those of cytotoxic agents, immunotherapies for pediatric cancers have the potential to offer the hope of cure to children with relapsed cancers and to reduce the acute and long-term toxicities from cancer treatment (Maude et al. 2018). While the broad class of cancer immunotherapies includes the basic tenets of targeting or utilizing the host immune system to effect an antitumor response, there is a wide variability to what is considered in this class. Here we divide immunotherapies into (*a*) therapies that amplify the endogenous host antitumor response, such as immune checkpoint inhibitors; (*b*) protein therapeutics that facilitate host immune responses toward tumor-specific cell surface molecules, such as monoclonal antibodies and bispecific antibodies; (*c*) protein therapies that capitalize on the specificity of antibodies to deliver potent drugs or radiation selectivity to tumors, such as antibody-drug conjugates (ADCs); and (*d*) cellular therapies that facilitate host immune responses toward tumor-specific cell surface molecules, such as chimeric antigen receptor (CAR) T cells. While the developmental origins and limited mutation burden of childhood tumors may limit the clinical effectiveness of some classes of immunotherapies such as checkpoint inhibitors, others such as CAR T cells have proven to be especially effective given the persistence of differentially expressed, lineage-specific cell surface molecules. Here we discuss the current and future use of these classes of immunotherapies in childhood cancers, the current challenges of their utilization, and future directions of pediatric immunotherapeutic discovery and development.

## ENGAGING THE ADAPTIVE IMMUNE SYSTEM

T cells provide a major mechanism for immune surveillance and tumor eradication; however, T cells can become tumor tolerant or exhausted, limiting their cytotoxic effects (Pauken & Wherry 2015). In 2018, the Nobel prize in Physiology or Medicine was awarded for the discovery that certain proteins on T cells such as cytotoxic T lymphocyte antigen 4 (CTLA-4) and programmed death receptor 1 (PD-1) can inhibit the antitumor effects of the host adaptive immune response following engagement of ligands on tumor cells such as programmed death receptor ligand 1 (PD-L1) (Topalian et al. 2015). Collectively, these studies led to the clinical development of several antibodies that block these host-tumor immune checkpoints and that have revolutionized the treatment of several adult cancers such as renal cell carcinoma, lung cancers, and melanoma (Garon et al. 2015,Motzer et al. 2015, Snyder et al. 2014). In contrast, immune checkpoint inhibition, which to date has been studied in a much more limited manner (Ansell et al. 2015, Bekoz et al. 2017, Blumenthal et al. 2016, Foran et al. 2017, Merchant et al. 2016), but with several ongoing trials (Kabir et al. 2018), has not yet demonstrated clinical success in common sporadic pediatric solid tumors. The most comprehensive pediatric clinical example to date is a recent phase I trial of the CTLA-4-blocking antibody ipilimumab, where no objective responses were observed in 33 patients, including 12 patients with pediatric melanoma and 17 patients with sarcoma, despite a pharmacokinetic and toxicity profile of the drug that was comparable to adult patients (Merchant et al. 2016). This lack of response to immune checkpoint inhibitors for most childhood cancers is likely a result of their overall lower immunogenicity compared to most adult tumors, which is in large part most likely derived from their lower mutational burden and limited neoantigen presentation (Chalmers et al. 2017).

Significant clinical responses to immune checkpoint inhibition have occurred in a small well-defined subset of childhood cancers including classical Hodgkin lymphomas (cHLs) (Ansell et al. 2015, Bekoz et al. 2017, Foran et al. 2017, Green et al. 2010, Haverkos et al. 2017) and tumors arising from children with germline biallelic mismatch repair deficiency (bMMRD) (Bouffet et al. 2016, Campbell et al. 2017). In both of these tumor histotypes, response to these agents can be best understood by considering aspects of their underlying tumor biology. cHLs are a B cell malignancy of adolescents and young adults that are histologically defined by a limited number of pathognomonic Reed-Sternberg cells that amass a large immune infiltrate with little evidence of antitumor activities (Kuppers 2009). Over one third of cHLs have been found to harbor a somatic gain of the *PD-L1* and *PD-L2* gene locus at chromosome 9q24.1 with resulting overexpression of the PD-L1 and PD-L2 checkpoint proteins (Green et al. 2010). Furthermore, the Epstein-Barr Virus, which is a key driver of cHL tumorigenesis, has been found to increase PD-L1 expression (Green et al. 2012). Taken together, these complementary mechanisms of PD-L1 overexpression in cHL lend a plausible biological mechanism to both the inactive inflammatory infiltrate commonly seen in these tumors and the impressive clinical response of these tumors to immune checkpoint blockade (Ansell et al. 2015, Bekoz et al. 2017, Foran et al. 2017, Green et al. 2010, Haverkos et al. 2017). In fact, pembrolizumab has recently been approved by the FDA (US Food and Drug Administration) for the treatment of cHL in pediatric patients. However, this approval was based on a trial that did not include pediatric patients (KEYNOTE-087; https://www.clinicaltrials.gov/ identifier NCT02453594) (Chen et al. 2017) and thus was based on an extrapolation of the 69% response rate from the 210 adult patients on this trial.

bMMRD is a rare but highly penetrant cancer predisposition syndrome that arises from homozygous germline mutations in one of the mismatch repair genes *PMS2, MLH1, MSH2,* or *MSH6* (Wimmer & Kratz 2010). Individuals with the bMMRD syndrome all develop cancer, typically within the first two decades of life, most commonly arising in the gastrointestinal tract, brain, or bone marrow. Tumors arising in individuals with germline bMMRD harbor by far the highest mutational burden among all human cancers (Campbell et al. 2017, Shlien et al. 2015). Thus, not surprisingly, these tumors have proven to be exquisitely responsive to checkpoint blockade in early clinical testing (Bouffet et al. 2016), and an ongoing clinical trial is evaluating the use of these therapies for children with bMMRD syndrome who develop cancer (NCT02992964).

Checkpoint inhibitors may also offer some utility in pediatric cancer clinical care in combination with both chemotherapy and radiotherapy (Herter-Sprie et al. 2016), as well as other targeted therapies (Goel et al. 2017, Hu-Lieskovan et al. 2015), including immunotherapies that capitalize on the differential expression of cell surface molecules such as ADCs and CAR T cells (Muller et al. 2015, Rios-Doria et al. 2017, Yoon et al. 2018). Additional utility from immune checkpoint therapies may also be realized in children with relapsed cancers, which often have a significantly higher mutation burden (Padovan-Merhar et al. 2016), and future clinical trials of immune checkpoint inhibitors will seek to determine whether such patients will experience a response to a single agent or combination immune checkpoint inhibition. Innovative technologies like profiling of circulating tumor DNA may also help predict which children with relapsed cancers may benefit from these therapies in real time (Weiss et al. 2017).

## TUMOR-TARGETING ANTIBODIES OR TUMOR-REDIRECTED T CELLS

**Figure 1:**
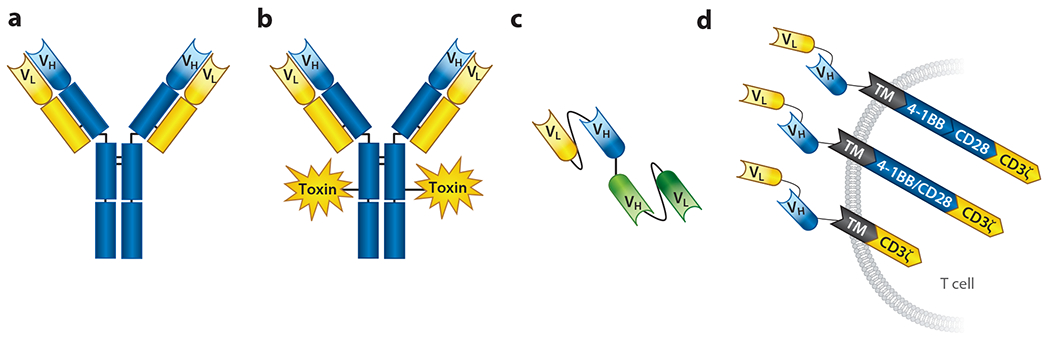
The diversity of immune-based therapies utilized to target pediatric cancers. Multiple immunotherapies have proven to be effective in pediatric cancers, including (*a*) monoclonal antibodies, (*b*) antibody-drug conjugates, (*c*) bispecific T cell-engaging antibodies with tumor-specific (*top*) and CD3-specific (*bottom*) single-chain variable fragments, and (*d*) CAR T cells, which have progressed from first-generation constructs (*bottom*) to second- (*middle*) and third-generation (*top*) molecules. Abbreviations: CAR, chimeric antigen receptor; TM, transmembrane region; V_H_, variable heavy chain; V_L_, variable light chain.

Advances in phage display and in the engineering of specific protein binders has enabled an increasing number of lineage-specific differentially expressed cell surface molecules on pediatric tumors to be targeted with an array of immunotherapeutics (Figure 1). These proteins can be used to recruit host immune cells to tumors, selectively deliver potent cytotoxic drugs or radiotherapeutics, or specifically target tumor-specific antigens with therapies such as CAR T cells engineered onto host immune cells, all of which are potentially applicable to a diverse set of childhood cancers.

## TARGET SELECTION: THE TUMOR-NORMAL TISSUE CONUNDRUM

### New Approaches to Antigen Discovery in Pediatric Cancer Immunotherapy

Until recently, a large majority of cell surface molecules that have been targeted with immune-based therapies across pediatric malignancies were either molecules that have been known for decades to be highly expressed on tumors, such as the disialoganglioside GD2 on neuroblastomas (Katano et al. 1983, Schulz et al. 1984) or CD19 on B cell leukemias, or molecules that have trickled down from adult oncology clinical trials, such as ERBB2 and CD20 (Sliwkowski & Mellman 2013). However, recently we and others have capitalized on the availability of large genomic, transcriptomic, and proteomic profiling data sets from tumors and normal tissues to discover and develop new immunotherapeutic cell surface targets specifically aimed at pediatric tumors (Bosse et al. 2017; Heitzeneder et al. 2019; Orentas et al. 2012, 2014).

Some important questions have arisen alongside these ongoing efforts to translate new cell surface molecules into safe and efficacious immune-based therapies. First, what is a safe tumor-normal tissue expression differential and does this threshold differ depending on the type of immunotherapy being utilized? Several relevant on-target, off-tumor side effects have recently been appreciated in the field of pediatric cancer immunotherapy. The GD2 disialoganglioside is abundantly expressed on most neuroblastomas (Schulz et al. 1984) and other pediatric and adult cancers (Chang et al. 1992, Cheresh et al. 1986), but it is also found on peripheral nerve fibers and in the central nervous system (CNS). GD2 expression on the former leads to significant levels of pain usually requiring on-demand opiates in children who receive the GD2-targeting chimeric monoclonal antibody dinutuximab (Suzuki & Cheung 2015, Yu et al. 2010). Interestingly, initial targeting of GD2 with CAR T cells has not resulted in comparable pain morbidity or any other CNS toxicity, but there has also been a paucity of antitumor activity (Heczey et al. 2017, Louis et al. 2011, Pule et al. 2008). However, more potent GD2-redirected CAR T cells that have been recently developed may be associated with neurotoxicity in preclinical animal models, but it remains unclear if these toxicities are the result of CNS GD2 targeting, an off-target effect against a different ganglioside, cytokine release syndrome (CRS), or nonspecific T cell killing in these murine models (Majzner et al. 2018, Mount et al. 2018, Richman & Milone 2018, Richman et al. 2018).

Targeting of L1CAM(CD171) with CART cells in neuroblastoma has also provided important lessons on the tumor-normal tissue expression threshold. L1CAM is overexpressed on neuroblastomas and many other pediatric and adult solid tumors with limited normal tissue expression, and a relatively tumor-restricted CE7 epitope of L1CAM has been the recent focus of targeting with CAR T cells (Hong et al. 2014). Robust preclinical safety testing in nonhuman primates expressing the identical CE7 L1CAM epitope in a similar normal tissue distribution to humans did not reveal any evidence for on-target, off-tumor toxicities at doses 100 times those given to humans (Kunkele et al. 2017). However, an initial phase I clinical trial in humans for recurrent/refractory neuroblastoma (NCT02311621) revealed the development of clinically relevant, albeit transient, skin rash and hyponatremia, potentially resulting from on-target, off-tumor CAR T cell L1CAM targeting in the skin, kidney, or pituitary gland (Pinto et al. 2018).

A final important example comes from the experience in targeting HER2 (human epidermal growth factor receptor 2) with CAR T cells. The first human CAR T cell trial targeting HER2 at the National Cancer Institute (NCI) resulted in respiratory collapse and death in a single patient treated with a trastuzumab-based CAR (Morgan et al. 2010). This was initially deemed by the researchers to be due to on-target, off-tumor CAR T cell activity against HER2 on normal lung epithelium. However, this patient was administered a CAR T cell dose that was much higher than what has been deemed the safe dose of CD19 CAR T cells and was also administered with exogenous IL-2. In retrospect, the clinical syndrome appears more consistent with CRS (Lee et al. 2019), which at the time was poorly understood. Recently, researchers at the Baylor College of Medicine have tested HER2 CAR T cells in a carefully designed dose escalation study using an alternative anti-HER2 binder in patients with both sarcomas and gliomas and have demonstrated both the efficacy and safety of this approach, with no signs of any off-tumor, on-target toxicities (N. Ahmed et al. 2015, 2017).

These recent preclinical and clinical experiences with CARs in pediatric solid tumors are a stark reminder of the importance of and difficulties in finding truly tumor-specific antigens for immunotherapeutic targeting. Despite rigorous preclinical toxicity screening in relevant animal models, trials should be carried out with the utmost safety considerations for these potent new cancer therapies.

Related questions have also arisen concerning how many absolute cell surface molecules are required to dictate robust efficacy for different types of immunotherapeutics and how homogeneous antigen expression needs to be to avoid the development of immune escape. Some recent studies have shown that homogeneous cell surface molecule expression needs to be high for robust CART cell activity (Fry et al. 2018, Walker et al. 2017); however, how this compares to other immune-based therapies is unclear. ADC-and radioconjugate-based therapies in particular may be more tolerant of the common solid tumor cell antigen heterogeneity due to bystander cytotoxic effects induced by intratumoral diffusion of payload (Golfier et al. 2014, Ogitani et al. 2016).

Finally, research into the role of gene splicing and posttranslational modifications in generating cancer-specific cell surface molecules that are safe to target remains in early stages for pediatric cancers. One important example is the CD44v6 isoform that is expressed in an array of adult epithelial cancers and pediatric and adult hematologic cancers (Amirghofran et al. 2016, Casucci et al. 2013, Magyarosy et al. 2001). While CD44 is widely expressed in human tissues, expression of the CD44v6 isoform is much more tumor restricted, creating the opportunity to safely target this molecule with several classes of immune-based therapies (Casucci et al. 2013, Haylock et al. 2017, Heider et al. 2004, Mortensen et al. 2018). A comprehensive survey of the pediatric cancer RNA splicing landscape and careful comparison with normal pediatric tissue isoform expression may reveal several new tumor-specific cell surface molecules to target with these potent therapies (Kahles et al. 2018).

### Monoclonal Antibodies and Antibody-Dependent Cellular Cytotoxicity

Monoclonal antibodies were the first type of immune-based therapy to show robust clinical efficacy in human trials (Figure 1a). The CD20-targeting monoclonal antibody rituximab has proven efficacy in pediatric non-Hodgkin lymphoma (Meinhardt et al. 2010). Perhaps more significant for the field of pediatric oncology, anti-GD2 antibodies such as dinutuximab (ch14.18) and naxitamab (hu3F8) have proven effective in the treatment of neuroblastoma, a solid tumor that occurs almost exclusively in children (Kushner et al. 2018, Yu et al. 2010). Dinutuximab is the first FDA-approved anticancer monoclonal antibody with an exclusive pediatric indication, and given concurrently with cytokines in the maintenance phase of therapy, it has become part of the standard of care for newly diagnosed neuroblastoma (Yu et al. 2010). Further, dinutuximab administered with irinotecan and temozolomide has become a first-line salvage therapy at the time of neuroblastoma relapse, achieving objective response rates of approximately 50% (Mody et al. 2017). Efforts are now focused on studying dinutuximab in combination with induction chemotherapy for newly diagnosed neuroblastoma. GD2 is also robustly expressed on other pediatric and adult cancers such as sarcomas (Chang et al. 1992), melanomas (Tsuchida et al. 1987), and small-cell lung cancers (Cheresh et al. 1986); thus, there are also efforts to extend the utility of anti-GD2 antibodies to these other cancers.

Some monoclonal antibodies such as rituximab and dinutuximab exert their tumor-cytotoxic effects via antibody-dependent cellular cytotoxicity, whereby the dual engagement of the antibody with a tumor-specific cell surface molecule and immune effector cells (typically natural killer cells and macrophages) via Fc receptor binding activates the immune effector cell, which then kills the tumor cell. Several antibodies aimed at inhibiting key pediatric cancer-associated signaling pathways have also been studied in an array of pediatric tumors but have not been nearly as effective as rituximab and dinutuximab; these include the IGF1R (insulin-like growth factor 1 receptor)-targeting antibodies cixutumumab and teprotumumab (R1507) (Malempati et al. 2019; Pappo et al. 2011, 2014; Wagner et al. 2015; Weigel et al. 2014) and the HER2-targeting antibody trastuzumab in osteosarcoma (Ebb et al. 2012). Trastuzumab has not been as effective as rituximab and dinutuximab despite being very effective in adult malignancies with *ERBB2* amplification (Slamon et al. 2001). B7-H3 (CD276), an important immune checkpoint family member, is also robustly differentially overexpressed on several pediatric (and adult) solid tumors, and several B7-H3 antibodies are currently being evaluated in the clinic (M. Ahmed et al. 2015, Loo et al. 2012, Picarda et al. 2016).

### Antibody-Drug Conjugates and Radioconjugates

Antibodies conjugated to drugs or radioactive molecules offer a potentially more potent mechanism to capitalize on the tumor cell specificity of antibodies to effect targeted and robust cytotoxicity in both liquid and solid pediatric malignancies (Figure 1b). Several ADCs have been clinically efficacious in pediatric leukemias and lymphomas, largely due to the translation of effective adult ADCs to pediatric-equivalent histotypes (Connors et al. 2018, Hills et al. 2014, Kantarjian et al. 2016, Younes et al. 2010). Most impressively, brentuximab vedotin, which consists of a CD30-targeting antibody conjugated to the tubulin binder monomethyl auristatin E, was shown to be safe and effective for children with relapsed/refractory CD30^+^ Hodgkin lymphoma or systemic anaplastic lymphoma (Locatelli et al. 2018). Similarly, gemtuzumab ozogamicin and inotuzumab ozogamicin, antibodies respectively targeting CD33 and CD22 that are each conjugated to the payload ozogamicin (an antitumor, antibiotic calicheamicin derivative), have shown efficacy in pediatric acute myeloid leukemia (AML) and acute lymphocytic leukemia (ALL), respectively (Aplenc et al. 2008, Bhojwani et al. 2018, de Vries et al. 2012, Gamis et al. 2014, Guest et al. 2017 Rytting et al. 2014, Tarlock et al. 2016). B7-H3 (CD276) antibodies may also be well suited for ADCs or immunoradioconjugates, with promising initial preclinical and clinical studies across several pediatric solid tumors, including those occurring in the CNS (Kramer et al. 2010, Modak et al. 2005, Onda et al. 2004, Seaman et al. 2017, Souweidane et al. 2018). Finally, preclinical models have proven the robust efficacy of using ADCs to target the tumor-specific molecules CD56, GPC2, and ALK recently discovered on neuroblastomas and other pediatric solid tumors (Bosse et al. 2017, Feng et al. 2016, Sano et al. 2019, Wood et al. 2013). Given the potency of ADCs and their potential tolerance of more heterogeneous antigen expression (Golfier et al. 2014, Li et al. 2016, Ogitani et al. 2016), these therapeutics maybe ideally suited for pediatric solid tumors. Clearly more research on tumor-specific antibodies as carriers of potent drug and radiotherapeutic payloads is imperative in pediatric immuno-oncology. Understanding how the internalization kinetics of antibodies differs in tumors versus normal tissues also remains a critical knowledge gap for this class of immunotherapeutics.

### Bispecific T Cell-Engaging Antibodies

Much recent effort has also been focused on capitalizing on the specificity of monoclonal antibodies to engineer bispecific protein therapeutics that can induce a robust tumor-immune synapse by recruiting host T cells to tumors. These so-called bispecific antibodies that simultaneously bind tumor-specific antigens and T cells via their CD3 receptor (Figure 1c) can induce robust T cell activation and tumor killing. Several bispecific antibodies have been developed and are clinically efficacious in pediatric cancers, most impressively in leukemias with the anti-CD19/anti-CD3-targeting bispecific antibody blinatumomab (Gore et al. 2018, von Stackelberg et al. 2016). Development of this class of immunotherapies has also been limited to date in pediatric oncology and it will be imperative to understand if other pediatric tumor–specific antibodies can provide adequate tumor targeting and T cell recruitment when engineered into bispecific constructs (Heitzeneder et al. 2019; Loo et al. 2012; Pappo et al. 2011, 2014; Yu et al. 2010). One additional major challenge facing the pediatric cancer immunotherapy field that is especially relevant to this class of therapies is the development of suitable humanized animal models to enable robust preclinical efficacy testing.

### Chimeric Antigen Receptor T Cell-Based Immunotherapies

CAR T cells have been developed to combine the cytolytic capacity of host T cells with the specificity of monoclonal antibodies in an MHC-independent manner. Similar to other antibody-based therapies, CARs can target any molecule expressed on the surface of tumor cells, as the receptor consists of an antigen-binding domain, most often the single-chain variable fragment of a monoclonal antibody, fused to a transmembrane domain and intracellular signaling endodomains, including CD3ζ and a costimulatory domain such as CD28 or 4-1BB (Figure 1d) (Labanieh et al. 2018). To date, the most well-developed clinical CAR T cell programs have been in the B cell malignancies, as lineage-restricted targets such as CD19 and CD22 are found exclusively within the B cell compartment and B cell aplasia is manageable in the clinic with regular intravenous immunoglobulin administration.

CD19 CAR T cells have induced deep and durable remissions in a large proportion of B cell malignancies in patients of all ages, with the highest response rates thus far in trials of children and young adults with B cell ALL (B-ALL). Across several clinical trials with different CAR constructs, CD19 CAR T cells have induced remissions in 70–90% of children with relapsed and refractory B-ALL (Jacoby et al. 2018; Lee et al. 2015; Maude et al. 2014, 2018). These results are unparalleled for a phase I clinical trial in this disease (or in cancer in general), and they rapidly led to the first FDA approval of CD19-directed CAR T cells for B-ALL. Importantly, the first two published pediatric clinical trials of CD19 CARs in B-ALL illustrate both the impressive clinical efficacy of these therapeutics and the fact that not every CAR molecule is created equally, with a major divergence in long-term persistence seen between different CAR architectures (Lee et al. 2015, Maude et al. 2014). At the Children’s Hospital of Philadelphia, researchers studied the safety and activity of a CD19 CAR containing the 4-1BB costimulatory endodomain, achieving a complete response rate of 90% of infused patients after a single dose of CAR T cells, with a persistence of T cells in 68% of patients at six months after T cell infusion (Maude et al. 2014). Concurrently, researchers at the NCI conducted a phase I clinical trial of CD19 CART cells containing a CD28 costimulatory endodomain in pediatric and young adult patients with a reported response rate for B-ALL of 70%, but T cell persistence was not seen (Lee et al. 2015). Thus, this CD19-focused work has taught us important intricacies to CAR engineering: While CD19 CAR constructs containing either 4-1BB or CD28 costimulatory domains are capable of inducing clinical remissions in a majority of B-ALL patients, only those CARs containing 4-1BB signaling domains appear to induce the long-term persistence essential for durable remissions.

Although very strong preclinical activity portended the clinical successes of CD19 CART cells (Brentjens et al. 2007, Kochenderfer et al. 2010, Milone et al. 2009), the laboratory models did not predict the clinical toxicity observed in human trials. CD19 CAR T cell administration has been associated with CRS, in which patients develop a sepsis-like condition driven by abnormally high levels of circulating cytokines (Lee et al. 2019). Patients with severe CRS have extraordinarily high levels of serum IL-6, and thus the IL-6 receptor-blocking antibody tocilizumab can enact drastic clinical improvement (Maude et al. 2014). While tocilizumab has now been widely adopted to treat CRS, it has not been effective in preventing immune effector cell-associated neurotoxicity syndrome, a typically self-limited syndrome in which patients develop encephalopathy, delirium, aphasia, seizures, and other CNS toxicities (Lee et al. 2019).

Although CAR T cells have revolutionized the treatment of relapsed and refractory B-ALL, for pediatric solid tumors these therapies have thus far shown only limited benefit to a small number of patients. The first CAR T cells infused into a pediatric patient contained a GD2 CAR (with the same antigen-binding moiety as dinutuximab) (Louis et al. 2011, Pule et al. 2008). This construct, a first-generation CAR containing only the CD3ζ endodomain but no costimulatory molecule (Figure 1d), was tested in a phase I clinical trial of patients with relapsed and refractory neuroblastoma. In all, 3 of 11 (27%) treated patients achieved complete remission, but CAR T cell persistence was infrequent and all patients eventually suffered disease relapse. Furthermore, clinical trials using a GD2 CAR engineered to contain costimulatory domains to enhance persistence showed no objective responses, even when CAR T cells were given in combination with PD-1 checkpoint blockade (Heczey et al. 2017). Pediatric patients have also been treated with CAR T cells targeting HER2 (sarcomas and glioblastoma) and L1CAM (neuroblastoma), and definite signs of clinical activity have been observed in recent studies (N. Ahmed et al. 2015, Pinto et al. 2018). However, it is likely that with improved T cell engineering (Labanieh et al. 2018) CAR T cells will ultimately prove effective in some settings for children with solid and brain tumors. In fact, definitive evidence of the potential for engineered T cells to mediate antitumor responses in patients with solid tumors has recently come from a clinical trial of an engineered T cell receptor (TCR) targeting NY-ESO-1 (D’Angelo et al. 2018). Patients with synovial sarcoma were infused with autologous T cells transduced with the NY-ESO-1^c259^ TCR, resulting in a 50% objective response rate, including a complete response that lasted nine months in a patient with diffuse pulmonary metastases (D’Angelo et al. 2018).

Overall, CAR T cells have now been robustly established as an important modality in pediatric cancer treatment, highlighted by the significant and durable clinical responses in B-ALL. In the short term, CAR T cells will likely move into earlier phases of treatment for B-ALL to prevent the toxicities associated with standard high-dose chemotherapy and stem cell transplant and will likely be translated to other hematologic malignancies such as AML where relapsed disease remains fatal. Finally, as T cells clearly can traffic to and eradicate solid tumors, a major focus in the coming years will be to develop clinically effective CARs for pediatric solid and brain tumors.

### Acquired Resistance to Immunotherapies

**Figure 2:**
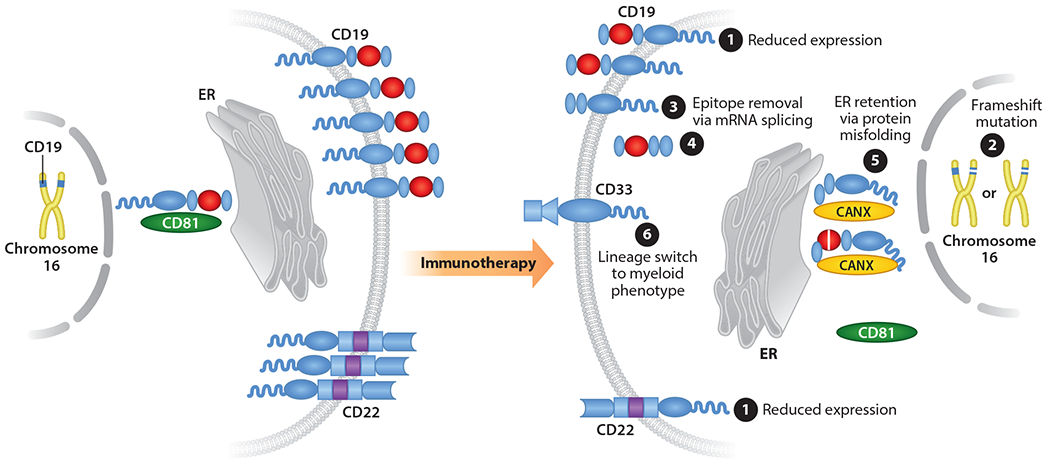
The diverse mechanisms of acquired resistance to CD19-targeted (*top*) and CD22-targeted (*bottom*) immunotherapies in pediatric B cell acute lymphoblastic leukemia. (❶) Selection for clones with lower CD19/CD22 expression. (❷) *CD19* frameshift mutations with or without deletion of the other *CD19* allele, leading to absent expression of the CD19 CAR-binding epitope. (❸, ❷) mRNA splicing removing the (❸) CD19-targeted epitopes or (❹) transmembrane domain epitopes. (❺) CD19 protein misfolding and endoplasmic reticulum (ER) retention due to altered mRNA splicing or in-frame insertions such that misfolded CD19 binds to ER chaperone proteins [i.e., CANX (calnexin)] instead of membrane-targeting CD81 molecules. (❻) Lineage switch to a myeloid phenotype with loss of CD19 expression.

Despite the immunotherapy revolution that has occurred in pediatric oncology over the last decade, resistance to even the most potent of these therapies has become an emerging clinical problem (Figure 2). As discussed above, CD19-redirected CAR T cell products have shown remarkable potency in pediatric hematologic malignancies. However, despite these clinical successes, as more patients have been treated with these therapies and their follow-ups extended, relapses have become common due to diverse mechanisms of antigen escape, all of which render the tumor cells undetectable to CAR T cells (Bagashev et al. 2018, Balducci et al. 2017, Braig et al. 2017, Jacoby et al. 2016, Rayes et al. 2016, Sotillo et al. 2015, Zoghbi et al. 2017). In the global registration trial for the CD19-targeting CAR T therapy tisagenlecleucel in B-ALL, 15 of the 16 relapses (94%) analyzed were CD19 negative (Maude et al. 2018). Additionally, a recent survey across several pediatric phase I/II clinical trials utilizing multiple CD19-redirected CAR constructs showed that up to 25% of children with B-ALL treated with a CD19 CAR T cell product ultimately suffered a CD19-negative relapse, accounting for a majority of the recorded relapses (Majzner & Mackall 2018). Similar rates of relapse with CD19 targeting in pediatric leukemias have been seen with the T cell-engaging bispecific antibody blinatumomab (Aldoss et al. 2017, Topp et al. 2014). The diverse mechanisms of CD19 downregulation under CD19 immunotherapeutic pressure are impressive, including deletion of the entire *CD19* genomic locus, acquisition of *CD19* frameshift mutations, alternative *CD19* mRNA splicing to remove the targeted epitopes or transmembrane domain, and disruption of CD19 trafficking to the cell membrane, as well as leukemia lineage switch to a myeloid phenotype with concurrent loss of CD19 expression (Figure 2) (Bagashev et al. 2018, Balducci et al. 2017, Braig et al. 2017, Jacoby et al. 2016, Rayes et al. 2016, Sotillo et al. 2015, Zoghbi et al. 2017). Immunotherapy-resistant *CD19* splice variants appear to be present at low levels in diagnostic samples, suggesting that CD19-targeting immunotherapies may simply select for these alternatively spliced CD19 isoforms lacking the targeted CD19 epitope rather than actively inducing the splice alterations themselves (Fischer et al. 2017).

Similar to CD19, CD22-directed immunotherapies for acute leukemias are also susceptible to low antigen density as a mechanism of therapy resistance, as observed under the selective pressures of both the CD22-targeting ADC inotuzumab ozogamicin (Paul et al. 2019, Shah et al. 2015) and CD22-directed CAR T cells (Fry et al. 2018). However, unlike CD19, the development of resistance to CD22-directed therapies has been associated with the selection of tumor subclones with low (but detectable) CD22 protein expression rather than the acquisition of *CD22* coding mutations or modulation of *CD22* mRNA expression or splicing (Figure 2) (Fry et al. 2018). The more robust homogeneous expression of CD19 compared to CD22 in pediatric acute leukemias (Shah et al. 2015) may be a cause of these differences in resistance mechanisms.

Given these recent findings, with the development of more efficacious CAR T cell products specifically targeting pediatric solid tumor cell surface molecules, it is imperative that we design trials with associated correlative biology studies to understand if the often heterogeneous cell surface expression in these tumors makes relapse more likely with low-antigen-expressing tumor clones. However, it remains unknown whether targeting different molecules will be as susceptible to antigen loss mechanisms as CD19-or CD22-directed therapies. Limited data for GD2 suggest that antigen loss may also occur in neuroblastoma relapses after treatment with the GD2-targeting antibody dinutuximab, but this has not been widely studied (Schumacher-Kuckelkorn et al. 2005, 2017). Targeting dual antigens on cancer cells may provide a solution to antigen loss. Several groups have recently initiated clinical trials of CD19/CD22 bispecific CART cell products in children with B-ALL (https://clinicaltrials.gov/ identifiers NCT03330691, NCT03241940, NCT03448393, and NCT03289455), which hold the promise of reducing the frequency of relapse driven by antigen loss.

It is also essential to understand if other classes of immunotherapies are as susceptible to antigen escape as CAR T cells, which again requires well-designed correlative biology studies in early-phase clinical trials. While ADCs appear to be reliant on homogeneous high antigen densities in the hematologic malignancies (Lamba et al. 2017, Olombel et al. 2016, Pollard et al. 2016), pediatric solid tumors may be less susceptible to the development of relapses of antigen-low variants after treatment with some ADCs, given the prevalence of bystander cell killing via intratumoral payload diffusion (Golfier et al. 2014, Ogitani et al. 2016). However, the development of ADC resistance is also a real possibility given their complex mechanism of action, which requires not only target binding on the cell surface but also internalization of the ADC, proper trafficking to the lysosome, lysosomal-mediated release of the payload, and sensitivity to the payload without the presence of drug efflux pumps (Garcia-Alonso et al. 2018, Walter et al. 2007).

Finally, with the large-scale genomic characterization of pediatric tumors coupled with the advent ofhigh-throughput technologies to identify unique binders directed at cell surface proteins, it has become feasible to target newly discovered cell surface molecules with relative ease, enabling the targeting of cell surface oncogenes. Whether targeting tumor-dependent molecules will help circumvent antigen-loss immune escape will also need to be studied in well-designed correlative biology studies as therapies targeting these molecules are translated to pediatric clinical care.

## CONCLUSIONS

Pediatric cancer immunotherapy has been revolutionized in the last decade, opening the door to cures for children with previously lethal diseases. Advances in the engineering of immune-based therapies have now created an effective pipeline and clinical framework to develop and study these therapies widely across pediatric tumor histotypes. Immunotherapy is clearly credentialed for childhood cancer, and the challenge now is the rapid discovery and development of optimal immunotherapeutic strategies, particularly for solid tumor and CNS malignancies.
